# Case Report of a 63-Year-Old Patient With Alzheimer Disease and a Novel *Presenilin 2* Mutation

**DOI:** 10.1097/WAD.0000000000000269

**Published:** 2018-08-17

**Authors:** Jennie L. Wells, Stephen H. Pasternak

**Affiliations:** * Department of Medicine, Division of Geriatric Medicine, Schulich School of Medicine and Dentistry, Western University; † St. Joseph’s Health Care London—Parkwood Institute; ‡ Molecular Medicine Research Group, Robarts Research Institute; § Department of Clinical Neurological Sciences, Schulich School of Medicine and Dentistry, University of Western Ontario, London, ON, Canada

**Keywords:** Alzheimer disease, *presenilin*, mutation

Early onset Alzheimer disease (EOAD) is a neurodegenerative dementing disorder that is relatively rare (<1% of all Alzheimer cases). Various genetic mutations of the presenilin 1 (*PSEN1*) and *presenilin 2* (*PSEN2*) as well as the amyloid precursor protein (APP) gene have been implicated. Mutations of *PSEN1* and *PSEN2* alter γ-secretase enzyme that cleaves APP resulting in increase in the relative amount of the more amyloidogenic Aβ42 that is produced.^1^

*PSEN2* has been less studied than *PSEN1* and fewer mutations are known. Here, we report a case of a 63-year-old woman (at the time of death) with the clinical history consistent with Alzheimer D, an autopsy with brain histopathology supporting Alzheimer disease (AD), congophylic angiopathy, and Lewy Body pathology, and whose medical genetic testing reveals a novel *PSEN2* mutation of adenosine replacing cytosine at codon 222, nucleotide position 665 (lysine replacing threonine) that has never been previously reported. This suggests that genetic testing may be useful in older patients with mixed pathology.

## CASE REPORT

**FIGURE 1:**
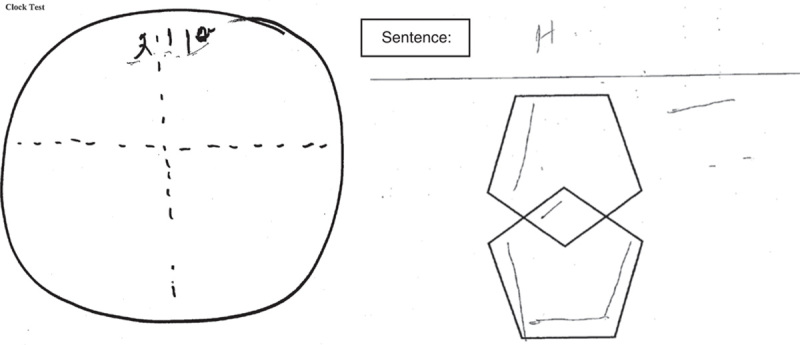
Visuospatial and executive function testing at age 58.

The patient was referred to our specialty memory clinic at the age of 58 with a 2-year history of repetitiveness, memory loss, and executive function loss. Magnetic resonance imaging scan at age 58 revealed mild generalized cortical atrophy. She is white with 2 years of postsecondary education. Retirement at age 48 from employment as a manager in telecommunications company was because family finances allowed and not because of cognitive challenges with work. Progressive cognitive decline was evident by the report of deficits in instrumental activities of daily living performance over the past 9 months before her initial consultation in the memory clinic. Word finding and literacy skills were noted to have deteriorated in the preceding 6 months according to her spouse. Examples of functional losses were being slower in processing and carrying out instructions, not knowing how to turn off the stove, and becoming unable to assist in boat docking which was the couple’s pastime. She stopped driving a motor vehicle about 6 months before her memory clinic consultation. Her past medical history was relevant for hypercholesterolemia and vitamin D deficiency. She had no surgical history. She had no history of smoking, alcohol, or other drug misuse. Laboratory screening was normal. There was no first-degree family history of presenile dementia. Neurocognitive assessment at the first clinic visit revealed a Mini Mental State Examination (MMSE) score of 14/30; poor verbal fluency (patient was able to produce only 5 animal names and 1 F-word in 1 min) as well as poor visuospatial and executive skills (Fig. 1). She had fluent speech without semantic deficits. Her neurological examination was pertinent for normal muscle tone and power, mild ideomotor apraxia on performing commands for motor tasks with no suggestion of cerebellar dysfunction, normal gait, no frontal release signs. Her speech was fluent with obvious word finding difficulties but with no phonemic or semantic paraphrasic errors. Her general physical examination was unremarkable without evidence of presenile cataracts. She had normal hearing. There was no evidence of depression or psychotic symptoms.

At the time of the initial assessment, her mother was deceased at age 79 after a hip fracture with a history long-term smoking and idiopathic pulmonary fibrosis. Her family believes that there is possible German and Danish descent on her father’s side. Her father was alive and well at age 80 at the time of her presentation with a history coronary artery disease. He is still alive and well with no functional or cognitive concerns at age 87 at the time of writing this report. Her paternal grandfather died at approximately age 33 of appendicitis with her paternal grandmother living with mild memory loss but without known dementia or motor symptoms until age 76, dying after complications of abdominal surgery. Her paternal uncle was diagnosed with Parkinson disease in his 40s and died at age 58. Her maternal grandmother was reported to be functionally intact, but mildly forgetful at the time of her death at age 89. The maternal grandfather had multiple myocardial infarctions and died of congestive heart failure at age 75. She was the eldest of 4 siblings (ages 44 to 56 at the time of presentation); none had cognitive problems. She had no children.

**FIGURE 2:**
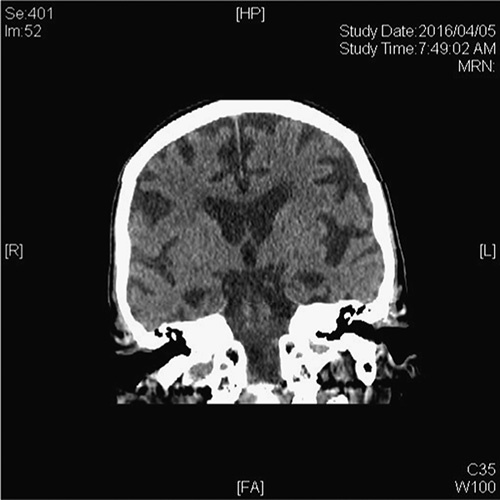
Coronal view, computed tomographic image, patient age 63, showing significant generalized atrophy and dramatic hippocampal atrophy.

Because of her young age and clinical presentation with no personality changes, language or motor change, nor fluctuations, EOAD was the most likely clinical diagnosis. As visuospatial challenges were marked at her first visit and poor depth perception developing over time, posterior cortical variant of AD was also on the differential as was atypical presentation of frontotemporal dementias. Without fluctuations, Parkinsonism, falls, hallucinations, or altered attention, Lewy Body dementia was deemed unlikely. After treatment with a cholinesterase inhibitor, her MMSE improved to 18/30, tested 15 months later with stability in function. Verbal fluency improved marginally with 7 animals and 3 F-words. After an additional 18 months, function and cognition declined (MMSE=13/30) so memantine was added. The stabilizing response to the cholinesterase inhibitor added some degree of confidence to the EOAD diagnosis. In the subsequent 4 years, she continued to decline in cognition and function such that admission to a care facility was required with associated total dependence for basic activities of daily living. Noted by family before transfer to the long-term care facility were episodic possible hallucinations. It was challenging to know if what was described was misinterpretation of objects in view or a true hallucination. During this time, she developed muscle rigidity, motor apraxias, worsening perceptual, and language skills and became dependent for all activities of daily livings. At the fourth year of treatment, occasional myoclonus was noted. She was a 1 person assist for walking because of increased risk of falls. After 1 year in the care home, she was admitted to the acute care hospital in respiratory distress. CT brain imaging during that admission revealed marked generalized global cortical atrophy and marked hippocampal atrophy (Fig. 2). She died at age 63 of pneumonia. An autopsy was performed confirming the cause of death and her diagnosis of AD, showing numerous plaques and tangles with congophilic amyloid angiopathy. In addition, there was prominent Lewy Body pathology noted in the amygdala.

Three years before her death informed consent was obtained from the patient and family to perform medical genetic testing for EOAD. The standard panel offered by the laboratory was selected and included *PSEN1*, *PSEN2*, APP, and apoE analysis. Tests related to genes related to frontotemporal dementia were not requested based on clinical presentation and clinical judgement. This was carried out with blood samples and not cerebrospinal fluid because of patient, family, and health provider preference. The results revealed a novel *PSEN2* mutation with an adenosine replacing cytosine at nucleotide position 665, codon 222 [amino acid substation of lysine for threonine at position 221 (L221T)]. This *PSEN2* variant was noted to be novel to the laboratory’s database, noting that models predicted that this variant is likely pathogenic. The other notable potentially significant genetic finding is the apoliprotein E genotype was Є_3/4_.

## DISCUSSION

β-amyloid (Aβ) is a 38 to 43 amino acid peptide that aggregates in AD forming toxic soluble oligomers and insoluble amyloid fibrils which form plaques. Aβ is produced by the cleavage of the APP first by an α-secretase, which produces a 99 amino acid C-terminal fragment of APP, and then at a variable “gamma” position by the γ-secretase which releases the Aβ peptide itself. It is this second γ-cleavage which determines the length and therefore the pathogenicity of the Aβ peptides, with 42 amino acid form of Aβ having a high propensity to aggregate and being more toxic.

The γ-secretase is composed of at least 4 proteins, mAph1, PEN2, nicastrin, and *presenilin*. Of these proteins, *presenilin* has 2 distinct isoforms (*PSEN1* and *PSEN2*), which contain the catalytic site responsible for the γ-cleavage. *PSEN* mutants are the most common genetic cause of AD with 247 mutations described in *PSEN1* and 48 mutations described in *PSEN2* (Alzgene database; www.alzforum.org/mutations). *PSEN2* mutations are reported to be associated with AD of both early onset and variable age onset as well as with other neurodegenerative disorders such as Lewy Body dementia, frontotemporal dementia, Parkinson dementia, and posterior cortical atrophy.^2^–^4^ In addition, *PSEN2* has associations with breast cancer and dilated cardiomyopathy.^3^

*PSEN2* mutants are believed to alter the γ-secretase cleavage of APP increasing the relative amount of the more toxic Aβ42. The mean age of onset in *PSEN2* mutations, is 55.3 years but the range of onset is surprisingly wide, spanning 39 to 83 years. Over 52% of cases are over 60 years. All cases have extensive amyloid plaque and neurofibrillary tangles, and many have extensive alpha-synuclein pathology as well.^5^

In considering the novelty of this reported *PSEN2* mutation, a literature search of Medline, the Alzgene genetic database of *PSEN2* and the Alzheimer Disease and Frontotemporal Dementia Mutation Databases (AD&FTMD) were completed (www.molgen.vib-ua.be/ADMutations). The mutation presented here (L221T) has never been described before.

**FIGURE 3:**
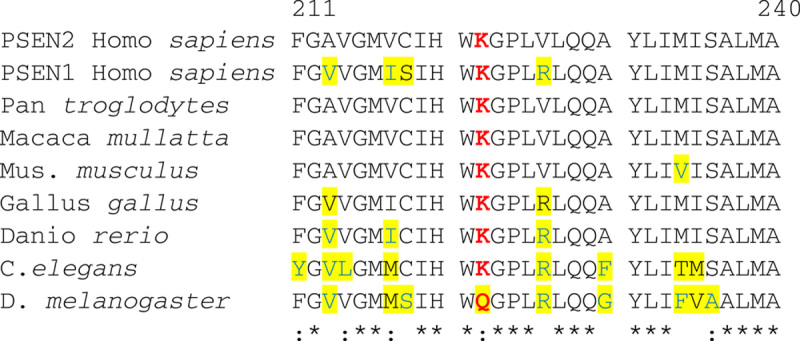
*PSEN2* sequence showing that lysine at position 221 is evolutionarily conserved. A 30 amino acid span of *PSEN2* (*Homo sapiens*) is shown covering K221, which is shown in red. Amino acids which differ for are highlighted. Sequences shown are *PSEN1* and *PSEN2* (from *H. sapiens*), *Pan troglodytes* (chimpanzee), *Macaca mullatta* (Rhesus Macaque monkey), *Mus musculus* (mouse), *Gallus gallus* (chicken), *Caenorhabditis elegans*, *Danio rerio* (zebrafish), *Drosophila melanogaster* (Drosophila; fruit fly). *Amino acids are identical across all samples; “:” conservative mutations with input sequence. Alignment performed using the tool MUSCLE.^6^ *PSEN* indicates *presenilin*.

Although this mutation has not been described, we believe that it is highly likely to be pathogenic. This mutation is not conservative, as it replaces a lysine residue which is positively charged with threonine which is an uncharged polar, hydrophilic amino acid. The mutation itself occurs in a small cytoplasmic loop between transmembrane domain 4 and 5, which is conserved in the *PSEN1* gene, and in *PSEN2* is highly conserved across vertebrates, including birds and zebrafish all the way to *Caenorhabditis elegans*, but differs in *Drosophila melanogaster* (fruit fly) (Fig. 3). We examined this mutation using several computer algorithms which examine the likelihood that a mutation will not be tolerated. Both SIFT (http://sift.bii.a-star.edu.sg) and PolyPhen-2.2.2 (HumVar) (www.bork.embl-heidelberg.de/PolyPhen) predicts that this variant is pathogenic. Interestingly, it is noted that *PSEN1* mutations after amino acid 200 develop amyloid angiopathy.^5^,^7^

This patient also had an additional risk factor for AD, being a heterozygote for the apoЄ4 allele. Among other mechanisms, its presence reduces clearance of Aβ42 from the brain and increases glial activation.^8^ Although the apoЄ4 allele is known to lower the age of onset of dementia in late onset AD, it has not been clearly shown to influence age of onset of EOAD in a limited case series.^9^ It should be noted that heterozygote state may have contributed to an acceleration of her course given the known metabolism of apoЄ4 and its association with accelerated cerebral amyloid and known reduction in age of onset.^10^

Given that there is no definite family history of autosomal dominant early onset dementia, it is likely that her *PSEN2* mutation was a new random event. With the unusually wide age of onset it is conceivable that one of her parents could still harbor this *PSEN2* mutation. The patient’s father, however, is currently 87 and living independently at the time of writing this manuscript, making him highly unlikely to be an EOAD carrier. Nonpaternity is an alternate explanation for the lack of known first-degree relative with EOAD; however, this is deemed unlikely by the family member who provided the supplemental history. Her mother died at age 79, so she could conceivably carry our mutation but we do not have access to this genetic material. Without extensive testing of many family members it would be impossible to speculate about autosomal recessive form of gene expression. In addition, the genetic testing requested was limited to *presenilins*, APP, and apoE mutations. Danish heritage may add Familial Danish dementia as a remote consideration; however, Familial Danish dementia has a much different clinical presentation with long tract signs, cerebellar dysfunction, onset in the fourth decade as well as hearing loss and cataracts at a young age.^11^ This disease has high autosomal dominant penetration which also makes it less likely in the patient’s context. This specific gene (chromosome 13) was not tested. The autopsy findings do not support this possibility. There are reports of Familial AD pedigrees in Germany, including a Volga pedigree with *PSEN141I* mutation in exon 5, but this is clearly separate from our mutation which is in exon 7. Our mutation was also not observed in a recent cohort of 23 German individuals with EOAD which underwent whole genome sequencing, but did find 2 carriers of the Volga pedigree. It is also possible that both the *PSEN2* mutation and the ApoE genotype contributed to her disease and early onset presentation. This case illustrates the multiple pathology types which occur in individuals bearing *PSEN2* mutations, and highlights the later ages in which patients can present with *PSEN2* mutations.^12^
